# Threat reversal learning and avoidance habits in generalised anxiety disorder

**DOI:** 10.1038/s41398-022-01981-3

**Published:** 2022-05-31

**Authors:** Clark Roberts, Annemieke M. Apergis-Schoute, Annette Bruhl, Magda Nowak, David S. Baldwin, Barbara J. Sahakian, Trevor W. Robbins

**Affiliations:** 1 Department of Psychiatry, University of Cambridge, Cambridge, UK; 2 Behavioural and Clinical Neuroscience Institute, University of Cambridge, Cambridge, UK; 3 Department of Psychology, University of Cambridge, Cambridge, UK; 4 Department of Neuroscience, Psychology and Behaviour, University of Leicester, Leicester, UK; 5 University Hospital of Zurich, Zurich, Switzerland; 6 Clinical and Experimental Sciences (CNS and Psychiatry), Faculty of Medicine, University of Southampton, Southampton, UK; 7 University Department of Psychiatry and Mental Health, University of Cape Town, Cape Town, South Africa

**Keywords:** Human behaviour, Physiology

## Abstract

Avoidance and heightened responses to perceived threats are key features of anxiety disorders. These disorders are characterised by inflexibility in dynamically updating behavioural and physiological responses to aversively conditioned cues or environmental contexts which are no longer objectively threatening, often manifesting in perseverative avoidance. However, less is known about how anxiety disorders might differ in adjusting to threat and safety shifts in the environment or how idiosyncratic avoidance responses are learned and persist. Twenty-eight patients with generalised anxiety disorder (GAD), without DSM co-morbidities, and 27 matched healthy controls were administered two previously established paradigms: Pavlovian threat reversal and shock avoidance habits through overtraining (assessed following devaluation with measures of perseverative responding). For both tasks we used subjective report scales and skin conductance responses (SCR). In the Pavlovian threat reversal task, patients with GAD showed a significantly overall higher SCR as well as a reduced differential SCR response compared to controls in the early but not late reversal phase. During the test of habitual avoidance responding, GAD patients did not differ from controls in task performance, habitual active avoidance responses during devaluation, or corresponding SCR during trials, but showed a trend toward more abstract confirmatory subjective justifications for continued avoidance following the task. GAD patients exhibited significantly greater skin conductance responses to signals of threat than controls, but did not exhibit the major deficits in reversal and safety signal learning shown previously by patients with OCD. Moreover, this patient group, again unlike OCD patients, did not show evidence of altered active avoidance learning or enhanced instrumental avoidance habits. Overall, these findings indicate no deficits in instrumental active avoidance or persistent avoidance habits, despite enhanced responses to Pavlovian threat cues in GAD. They suggest that GAD is characterised by passive, and not excessively rigid, avoidance styles.

## Introduction

Generalised anxiety disorder (GAD) is among the most common anxiety disorders, thought to affect roughly 4–7% of adults in the USA [1]. Epidemiological evidence indicates GAD is nearly twice as frequent in women [2], often being connected to adverse social factors in youth and showing high rates of comorbidity with other anxiety and mood disorders [3, 4]. Cardinal to the diagnosis is persistent uncontrollable worry, typically accompanied by restlessness, agitation, and muscle tension, which disrupt several domains of daily functioning [1]. Excessive worrying is exacerbated by the capacity to cognitively model aversive future outcomes and attach negative expectations about the likelihood and magnitude of those events, often occurring in a perseverative and debilitating fashion.

Perceived threats commonly elicit avoidance, which is implicitly defined as a core feature across anxiety disorders in the DSM5 [5] when it becomes detrimental to the individual. After innate responses are overcome following threats, threat-conditioned stimuli may then function as an adaptive reinforcer for idiosyncratic learned avoidance strategies [6]. However, a broad distinction can be drawn between passive avoidance, expressed by increased risk assessment and inaction to avoid adverse outcomes, and active avoidance responses, which involve learned proactive actions to avoid danger ([7]; [8]). Active avoidance is a form of goal-directed behaviour in which an instrumental action causes an omission of an aversive outcome. Such goal-directed behaviour can however become habitual when the conditioned stimuli directly elicit the response [9]. There have been recent demonstrations of such habitual avoidance responding both in animal [10] and human research when the aversive outcome is devalued, but behavioural avoidance responses persist. In particular, a proportion of OCD patients have exhibited perseverative habitual avoidance [11, 12], which may be a form of impaired inhibitory learning during extinction. Indeed, while research in OCD has indicated moderate evidence for abnormalities associated with the acquisition of aversively conditioned responses, there is more substantial support for impairments occurring during the extinction process [13, 14].

There are strong theoretical assumptions that aversive conditioning and extinction processes are inherent components of anxiety disorders, which lay at the foundation of currently effective behavioural treatments. However, there has been surprisingly little investigation of conditioning impairments that might be specific to GAD. One cross-sectional study of healthy controls and mixed anxiety states focused mainly on Pavlovian conditioning and extinction, including recall in a functional neuroimaging context- with evidence of generally blunted SCR but no differential impairment in conditioning to coloured image conditioned stimuli (CS) [15, 16]. A meta-analysis [17] found, as for adults, no evidence of impaired SCR fear conditioning or extinction in a group of anxious youths. However, the anxious youths did exhibit heightened responses to particular stimuli. A recent study used a large sample of GAD patients and found enhanced SCR in response to face stimuli but no deficit in differential threat conditioning [18]. However, this study did not investigate the possible inflexibility of aversive conditioning, when the source of anxiety changes and thus invalidates prior safety cues. Nor have there been extensive studies of instrumental avoidance conditioning in GAD and its flexibility or automaticity as may occur during the establishment of aversive habits.

To investigate possible elements of behavioural inflexibility or failures to update aversively conditioned associations in GAD, we focused on two tests that highlight those aspects: (i) a Pavlovian threat reversal-learning task in which the reversal phase evaluates the ability *to flexibly adjust* physiological responses [19] to changes in both threat and safety predictive stimuli; and (ii) a test of the *persistence* of active avoidance as a stimulus-response habit following devaluation of the shock [11]. Both paradigms have been shown to produce evidence of inflexible learning in patients with OCD [11, 12, 20], suggesting a contribution to compulsive symptoms in that disorder, although there was no evidence per se of enhanced aversive conditioning.

The Pavlovian threat reversal paradigm is more cognitively demanding than other threat extinction paradigms because valence shifts occur during the same experimental session, where reversals are unsignaled, and threat predictive cues are still present [19]. Further, this design allows for a better understanding of how faster behavioural and corresponding physiological shifts from threat to safety occur without acquiring generalised threat responses [21], which are consolidated and usually studied over longer periods. Recent work has indicated that this capacity may be aberrant in different anxiety disorders using the same reversal paradigm. Namely, individuals with OCD displayed impairments in differential learning [20]. While PTSD patients appeared to demonstrate successful differential learning, computational modelling showed a hypersensitivity to aversive prediction errors, scaling with the severity of PTSD symptoms [22]. This heterogeneity may reflect some of the differences among cognitive profiles in categorically unique anxiety disorders.

Cognitive profiles of GAD and OCD overlap in gradations of threat appraisal and hypervigilance [23], subjective scales measuring intolerance of uncertainty (IU) [24], and impairments in attentional control [25]. However, they differ in several behavioural characteristics, including the degree of compulsive responding and specificity and content of internally modelled anticipatory threats. The precise causal relationships between subjective anxiety, avoidance, and compulsions have been challenging to establish, given that compulsive behaviour can sometimes occur in the absence of anxiety, while still resembling an active form of avoidance. Further, some archetypal “anxiolytic” drugs, such as benzodiazepines, are not efficacious for OCD (for review: [26]). GAD is often expressed by the anticipation and subjective deliberation of more domain-general threats, which likely shapes different environmental avoidance strategies compared to more cue-specific diagnostic categories such as post-traumatic stress disorder (PTSD), social anxiety disorder (SAD), or OCD.

Thus, we sought to examine how (1) GAD patients differentiate and update physiological threat responses to dynamic ambiguous shifts in valence compared to controls and their response to safety signals; and (2) How GAD patients might differentially acquire active avoidance habits in previously established paradigms, and how those relate to subjective post hoc reports of anxiety. We hypothesised that GAD patients would show impaired SCR differential (CS+ minus CS−) during the reversal phase, reflecting an inability to process dynamic shifts in valence. Further, we hypothesised that GAD might show higher levels of active avoidance either during initial acquisition, or following devaluation, when compared to controls, but that the latter effects would be potentially less robust than findings from prior studies in OCD.

## Methods

### Subjects

**Fig. 1:**
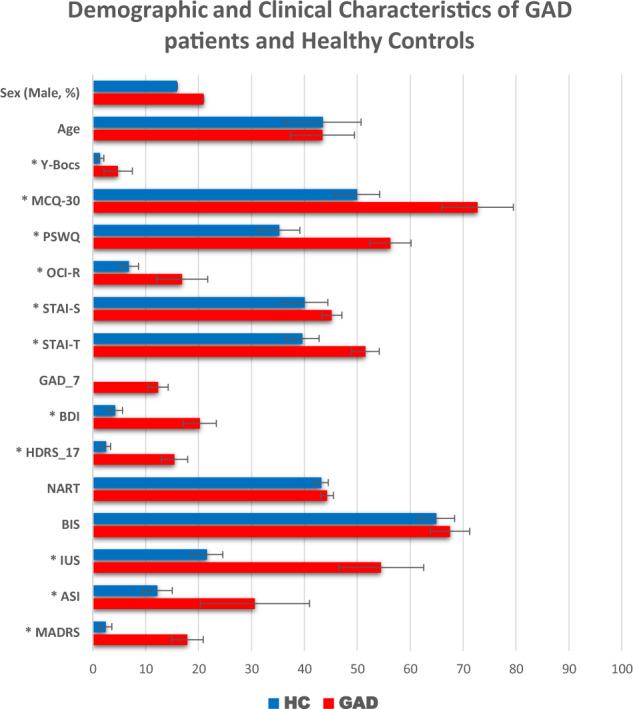
Significance denoted using asterisks on left (*p* < 0.05). Error bars show the 95% CI. Addiction Severity Index (ASI), Becks Depression Inventory (BDI), Behavioural Inhibition Scale (BIS), Hamilton Depression Rating Scale (HDRS_17), Intolerance of Uncertainty Scale (IUS), Metacognition Questionnaire (MCQ-30), Montgomery and Asberg Depression Rating Scale (MADRS), National Adult Reading Scale (NART), Obsessive Compulsive Inventory (OCI), State-Trait Anxiety Inventory (STAI), The Penn State Worry Questionnaire (PSWQ), Yale-Brown Obsessive Compulsive Scale (Y-BOCS).

For a significant difference in differential learning to be detected, a 20–25% decrease in learning strength would be expected in the GAD group as we previously found in OCD when averaged over the different stages [20]. A power calculation for a 25 percent decrease in mean GSR scores of 0.3, SD 0.1, would require a sample size of 25 in each group (25 Controls and 25 GAD patients) with Alpha set to 0.05 and Power of 80% [16]. In fact, we were able to test 28 carefully selected patients (7 male) with a psychiatrist-determined DSM-5 diagnosis of GAD, with no current co-morbidities, including MDD, and 27 healthy controls (4 male) matched for age, IQ, handedness, and years in education participated in this study (see Fig. 1 for demographic details). Individuals were excluded if they had any other significant co-morbid axis-1 disorder, current or previous neurological deficits, problems with eyesight, or history of excessive drug or alcohol abuse. Patients with GAD were recruited at the Mood and Anxiety Disorders Service in Southampton. Controls were screened using the MINI International Neuropsychiatric Interview. All non-English speakers were excluded. Both tasks were administered in a quiet testing room. Twenty-two participants in the GAD sample were currently prescribed psychotropic medication (mainly SSRIs). Due to non-completion and errors during testing, the final sample for the reversal task was 54 and the avoidance task was 53. This study received ethical approval from the Ethics Committee and all participants provided written informed consent.

### Task design

#### Study 1—Pavlovian threat reversal

**Fig. 2:**
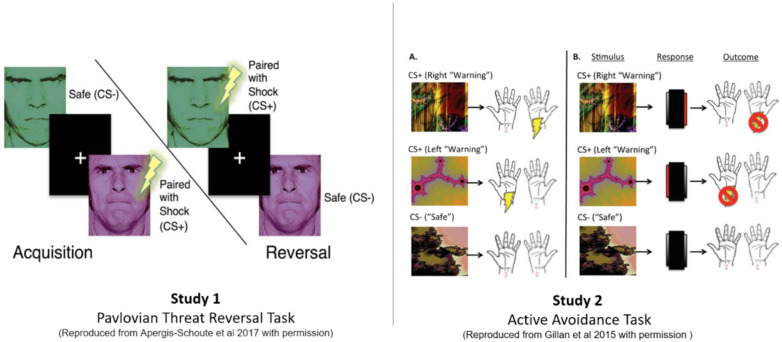
Task design. Study 1: Threat reversal paradigm. One of the faces co-terminated with a shock on 8 out of 24 trials. SCR analysis was done on the CS+ trials without shock. Aversive contingencies were then switched to the other face with the same shock ratio. Study 2: Pavlovian Avoidance Habit Acquisition. Pavlovian stimulus-outcome contingencies were 100% deterministic for 40 trials where different stimuli predicted shocks on either the left or right side. Participants were instructed they could cancel the shock corresponding to the otherwise aversive predictive stimuli by pressing a footbox on either the left or right side. One of the stimuli was not paired with shock and remained safe throughout the task.

For study 1, we employed a Pavlovian threat reversal paradigm [20] to examine differences in the capacity to flexibly adjust SCR responses to dynamic shifts in threat-safety CS contingencies paired with faces (see Fig. 2). This form of Pavlovian threat reversal learning requires extinction of the prior CS+, while the previous CS− stimulus acquires an aversive predictive value. The paradigm involved two stimuli consisting of coloured angry faces (Fig. 2), one of which was initially safe (CS−) and the other threat-conditioned with a shock (CS+), which were subsequently reversed in their valence, unsignaled to participants (see Fig. 2). Participants chose their own level of shock that was uncomfortable but not painful. CS’s were shown for 4 s followed by a 12 s inter-trial interval with a white fixation cross.

During acquisition, face A appeared 16 times with no shock (CS+) then 8 times with the shock (CS+US) while face B was not paired with shock over 16 trials. The contingencies were then switched during the reversal phase where face B appeared with shock on 8 trials (CS+ US) and 16 times without shock (CS+) while face A was not paired with shock (CS−, 16 trials). Trial types were pseudorandomised and faces were counterbalanced. Analysis only included trials without a shock, including the CS+ when shock was expected or CS− when shocks did not occur.

#### Study 2—Acquisition of habitual avoidance

For study 2 mentioned below, we adopted a paradigm from [11, 12]) (see Fig. 2), previously used to assess the acquisition of habitual avoidance responses in OCD, to examine if individuals with GAD showed similarities or differences in this type of experimentally induced active avoidance. Participants had shock electrodes attached to their left and right wrists and were trained with two corresponding CS’s that would predict shock, including one CS safety stimulus which was not paired with shock (see Fig. 1). They were told they could avoid the shock to the two threatening CS’s by pressing a corresponding pedal on the left or right side with their feet. Over four blocks (10 per CS), participants were over-trained to avoid the shocks and were shocked if they failed to respond. In the final block, electrodes were detached from the left wrist, and subjects were explicitly informed that avoidance responses were no longer required to avoid shock. The 5th block was a devaluation phase and defined as a habit test to see if perseverative avoidance responses persisted to the devalued CS despite being explicitly informed of its safety. Further, subjective retrospective ratings were taken on a visual analogue scale (VAS) from 0 to 100, showing their urge to perform, attempt to suppress habit responses, and degree of unpleasantness of shocks. Following testing, participants were asked to give subjective post hoc explanations about their urge to perform avoidance responses.

Skin conductance responses (SCR) from the left fingertips were measured on the onset of all aversive and safety CSs. Experiments were implemented using E-Prime, which was imported into AcqKnowledge for further analysis. SCR data were passed through a high pass filter of 0.05 hertz, removing low-frequency drift, as well as a low pass filter of 0.05 hertz to take out high-frequency scanner noise. SCR data were analysed by the peak difference within an 8.5 s interval following CS presentations. All SCR data were square-root transformed to correct for skew. SCR data were compared for CSs corresponding to Safe, Valued, and Devalued conditions within the task to assess any differences in skin conductance

## Results

### Study 1—Pavlovian threat reversal

#### GAD patients exhibit increased skin conductance during overall learning (Acquisition/Reversal) and phase (Early/Late) (Fig. 3)

**Fig. 3:**
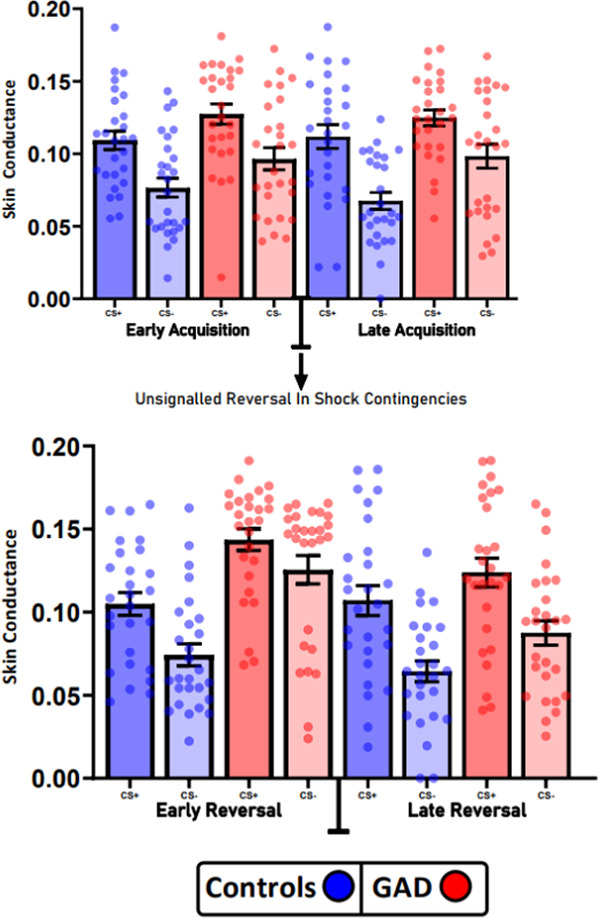
Analysis of SCR during Pavlovian threat reversal paradigm from early and late trials from the acquisition and reversal phases. Across all phases, GAD patients had higher overall SCR responses. Both groups were able to differentiate between the CS+ and CS−. However, relative to controls, GAD patients displayed impaired differentiation during the early reversal phase, indicating a slower adaptation to reversal contingencies. Error bars denote SEM.

A three-way ANOVA (Learning stage (Acquisition/Reversal, phase (Early/Late) and SCRs (CS+/CS−)showed a strong significant effect of group between Controls and GAD patients (F(1,52) = 21.25, *P* < 0.0001) and a significant 3-way interaction between learning stage, phase and group (F(1,52) = 4.502, *P* = 0.039), indicating significant differences in skin conductance responses to the CSCS+ and CS−, which were both angry faces. A table (Table S1) of the full ANOVA can be found in the Supplementary Material.

We also calculated the difference scores (CS+ minus CS−) per learning stage and phase to compare differential learning between groups using a two-way ANOVA which showed that averaged differential learning was not significantly different between Controls and GAD patients (F(1,52) = 2.184, *P* = 0.146) contrasting with the strong differential deficit found in OCD patients [20].

#### Additional trial by trial analyses of difference (CS+ minus CS−) scores (CS+ minus CS−) during Acquisition and Reversal

We performed an additional trial-by-trial analysis of differential learning (CS+ minus CS−) SCR scores during acquisition and reversal to reveal potential time linked differences between the groups that could have been masked by averaging over trials. This additional analysis (see Supplement for statistical analysis and accompanying figure—Supplementary Figure S1) revealed that GAD patients have reduced maintenance of differential learning during acquisition and are slower to reverse.

#### Healthy controls differentiate significantly between CS+ and CS− during each stage—a within-group analysis (Fig. 3)

To measure contingency knowledge, we directly contrasted SCRs to the CS+ and CS− within in each group per stage using paired T-tests [19, 20], which showed that controls differentiated significantly during each stage: early acquisition (*t*_26_ = 5.69, *P* < 0.005), late acquisition (*t*_26_ = 5.051, *P* < 0.005), early reversal (*t*_26_ = 4.197, *P* < 0.005) and late reversal (*t*_26_ = 4.851, *P* < 0.005).

#### GAD patients differentiate significantly between CS+ and CS− during all stages apart from early reversal—a within-group analysis (Fig. 3)

GAD patients also significantly differentiate between the CS+ and CS− during early acquisition (*t*_26_ = 2.827, *P* < 0.05), late acquisition (*t*_26_ = 2.838, *P* < 0.05) and late reversal (*t*_26_ = 4.29, *P* < 0.005), but showed a mild differentiation deficit in early reversal (*t*_26_ = 1.661, *P* = 0.11).

### Study 2—Acquisition of active avoidance habits

#### Behaviour

##### GAD patients and Controls did not differ in initial active avoidance acquisition or the expression of habitual avoidance responses following devaluation (Fig. 4)

A generalised logistic regression model was used to identify any difference between groups in terms of task performance prior to the devaluation phase. There was a trend toward superior response accuracy in healthy controls towards the valued stimulus (Z = 1.875, *p* = 0.061), but this was not significant. There was no significant difference between groups (Z = −1.314, *p* = 0.189) for response accuracy to the safe stimulus.

**Fig. 4:**
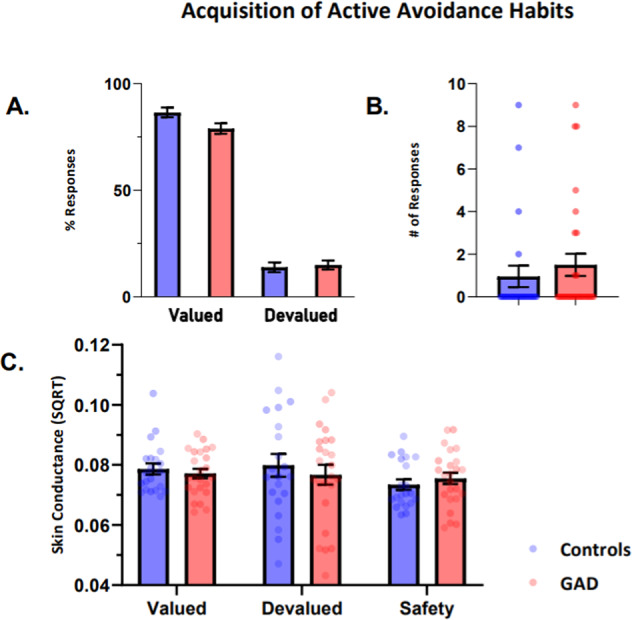
Acquisition of Avoidance Habits. **A** Percent of responses for controls and GAD patients during the learned avoidance task contrasting the valued and devalued phases. Behavioural responses did not differ during the valued or devalued stimulus presentation. **B** Number of continued avoidance responses made during the devaluation phase across groups. **C** Depicts SCR during avoidance task over different task conditions. Error bars denote SEM.

The majority of participants in both groups did not continue responding during the devaluation phase, with 5 healthy controls and 9 GAD patients continuing to respond (*χ*^2^ = 0.84, *p* = 0.359) (see Fig. 4). There was no significant between-group difference in the rate of responding to the devalued stimulus between control participants (mean = 1.64, SE = 0.66) and participants with GAD (mean = 1.50, SE = 0.52; F(1,51) = 0.028, *p* = 0.868). This was confirmed using a Mann–Whitney U test given the non-normal distribution of responses, which confirmed no significant differences between groups (U = 333, Z = 0.294, *p* = 0.772).

#### Skin conductance response

##### SCR was comparable among GAD individuals and Controls during Valued, Devalued, and Safety Conditions

Overall, there was no difference in SCRs between control participants (mean = 0.078, SE = 0.002) and GAD patients (mean = 0.076, SE = 0.001; F(1,132) = 0.59, *p* = 0.444), and no significant differences between any of the valued (mean = 0.078, SE = 0.001), devalued (mean = 0.079, SE = 0.003), or safe (mean = 0.075, SE = 0.001) conditions (F(2,132) = 1.61, *p* = 0.204). In addition, there was no significant interaction between group and condition (F(1,132) = 0.72, *p* = 0.489).

#### Subjective reports during habit test

**Fig. 5:**
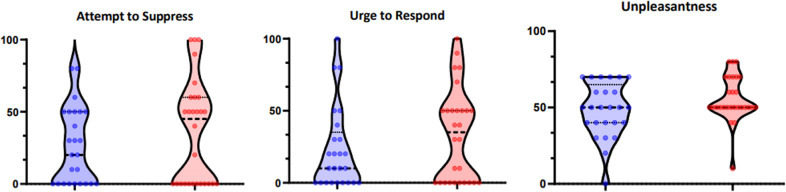
Post-hoc Subjective Ratings in Habit Task. Retrospective ratings from the Habit Acquisition paradigm on a visual analogue scale ranging from 0 to 100.

A Mann–Whitney test indicated that retrospective self-reported ratings (see Fig. 5) of unpleasantness out of 100 was not significantly different between healthy controls (median = 50) and GAD patients (median = 50) (U = 264.5, *Z* = 1.52, *p* = 0.131). Similarly, no significant difference was seen for the urge to respond for healthy controls (median = 20) and GAD patients (median = 45) (U = 310, *Z* = 0.70, *p* = 0.484). The same pattern was observed for the attempt to suppress, between healthy controls (median = 10) and GAD (median = 35) (U = 294.5, *Z* = 0.98, *p* = 0.327).

When subsequently asked to provide reasons why they may have continued to press both pedals in the final stage, some GAD patients showed a trend towards more intricate post hoc explanations (Table 1), indicating an increased magnitude of threat associated with their subjective accounts, with responses such as “*thought that was the best to do*”, “*seemed the natural response*”, “*safer to make sure didn’t get shocked*” and “*my mind was telling me to, after pressing it in the first couple of sessions*”.

**Table 1:** Retrospective qualitative ratings from participants who responded or felt an urge to respond during the devaluation phase of the habit test.

	No. of cases			
Subjective accounts	HC	GAD	*χ*^2^	*p*
Rationale “Felt I had to”	3	9	2.560	0.109
Accidental Slips “Mistake”	3	5	0.221	0.638
NA	17	14	3.029	0.082

*NA* not applicable.

## Discussion

Patients with GAD (screened to exclude major co-morbidities) exhibited significantly enhanced SCR responses to angry faces and a mild transient deficit in the early stages of aversive threat reversal, but no significant deficits in early differential aversive Pavlovian threat conditioning, instrumental active avoidance conditioning, or the establishment of aversive avoidance habits. Overall, these findings agree with previous findings of intact differential aversive conditioning in anxious patients [15] and also contrast with observations using similar paradigms for patients with OCD [11–13]. Therefore, GAD may be characterised by superior control over habitual compulsive responding than OCD.

The generally enhanced SCR response to angry faces shown by GAD patients was especially striking in view of the lack of any such difference for cued active avoidance conditioning, suggesting a special significance of the threatening face cues which has been noted in other contexts, for example, following transient down-regulation of serotonin by acute dietary tryptophan depletion in healthy volunteers (e.g. [27]). However, this enhanced response did not result in a change in early differential conditioning (neither enhancement nor deficit), despite the possibility of increased perceptual generalisation across the face stimuli in GAD. The enhanced SCR response to threatening faces, however, is consistent with what has been observed previously in GAD [18], and in youths with anxiety, at least in response to certain stimuli [17]. It may also account for the impairment in a trial-by-trial analysis of differential conditioning in late acquisition (Fig. S1).

There was a mild impairment in the reversal phase of updating the aversive valence of the facial stimuli in GAD, again possibly resulting from the generally elevated SCR responses, but this was only transient and could have resulted from the increased volatility associated with reversal learning and the GAD patients’ high intolerance of uncertainty scores (Table 1). The findings stand in contrast to these observed previously in OCD where the SCR response to the faces, if anything was reduced in relation to controls, but was also associated with impaired learning about the CS− (‘safety-signal’) not evident for GAD. Moreover, there was also a major, persistent deficit in both early and late Pavlovian reversal in OCD, with patients exhibiting generalised responding to both facial stimuli, likely connected to impaired safety signal processing [20]. Additional trial by trial analysis of differential threat learning and reversal (see Supplement) did reveal reduced strength in the maintenance of learning during acquisition and a slower return of CS+ versus CS− differentiation during reversal. These results indicate that although higher overall SCR responses to the angry faces in GAD patients do not cause a full deficit in differential acquisition or reversal, they do impact on how well learning can be maintained or how quickly it can be adjusted.

As in the case of Pavlovian conditioning, the GAD patients also exhibited no evidence of alterations in active avoidance conditioning. This was unlikely to have been due to task insensitivity, as patients with stimulant use disorder have previously been shown to be impaired in such active avoidance learning using the same test procedure [28]. Nor did they show any evidence of increased habit learning when one of the stimulus-outcome associations was devalued after an extended period of conditioning. This is again different from what has been observed in OCD where a substantial proportion of patients have been shown in two separate studies to exhibit persistent habit-like responding [11, 12]. This was also accompanied by persistent, irrational threat beliefs and increased urges to respond, which were not as prevalent in this GAD group.

Overall, it can be concluded that the elevated anxiety of GAD patients might be associated with enhanced SCR in response to certain provocative stimuli (angry faces), but does not in general cause obvious changes (either enhanced or impaired) in instrumental conditioning. Further, GAD patients displayed mild impairments in updating associations during reversal learning which could indicate a transient defensive inflexibility in volatile environments—potentially influencing the worry process at a subjective level. This comparison also makes it unlikely that the more robust pattern of effects seen in OCD for both instrumental and Pavlovian aversive conditioning results simply from higher levels of subjective anxiety and may relate more directly to the compulsive behaviours of OCD, or the overlap of compulsions and anxiety. Moreover, while SCR and subjective anxiety are often correlated, mapping their precise relationship has been difficult [29, 30].

One potential implication is that while the amplified acquisition of, and differentiation between, aversive, ecologically relevant learning signals might be a general aberrant feature of anxiety disorders, the singular phenotypic expression of GAD, compared to OCD, may be less characterised by active perseverative avoidance responses following outcome devaluation. Contrasting manifestations of avoidance strategies may parallel the distinction between passive avoidance, expressed by increased risk assessment and inaction to avoid negative outcomes, and active avoidance responses, which involve learned proactive actions to avoid danger ([7, 31]; [8]). Future work should assess the degree to which active and passive avoidance styles are differentially expressed in categorically distinct anxiety disorders.

This study had several limitations that should be highlighted. Co-morbid DSM diagnostic criterion were screened for, and while this may indicate a roughly disorder-specific effect, it may not be representative of the majority of co-morbid GAD cases. Most of the GAD patients were on medication, and while this presents a potential confound, it is conceivable that group differences without medication may have been even more robust. Despite the apparently contrasting experimental effects with (medicated) OCD, we did not formally compare these two groups in the same study, which future work should explore directly. This was a behavioural study, and no neural data were collected, which would have been helpful in providing further comparisons with OCD in the neural domain [12]. The Pavlovian threat reversal paradigm was previously conducted as part of an fMRI procedure rather than as a simple behavioural procedure, as here. However, the magnitude and patterning of the SCR responses in control subjects in the two studies appeared comparable, and the generally elevated SCR measures in GAD patients might have been expected to be even greater in the noisy and stressful environment provided by MRI. Given the complexity of safety signalling in the real world and the inherent variation in how aversive unconditioned stimuli are in laboratory settings, future work might hope to incorporate disorder-specific stimuli as well as more diverse multi-component cues across various contexts, including methodologies assessing semantic or conceptual generalisation [13]. Further work may assess how the temporal trajectory and amplification of Pavlovian vs instrumental biases dynamically connect with the etiology of particular avoidance strategies and the ontology of anxiety diagnostic classifications.

In conclusion, while GAD patients exhibited significantly greater skin conductance responses to signals of threat than controls, they did not exhibit the impaired acquisition and deficits in reversal learning shown previously by patients with OCD lacking learning of the safety signal in a Pavlovian conditioning paradigm. Thus, while threat generalisation may be present in GAD, it may not be simply due to an absence of stimulus-dependent safety signalling. Moreover, this patient group, again unlike OCD patients, did not show evidence of altered active avoidance learning or enhanced instrumental avoidance habits. Hence GAD is not characterised by the same behavioural patterns and habitual effects of Pavlovian and instrumental avoidance conditioning as in OCD—which are therefore unlikely to arise simply because of enhanced anxiety.

## Supplementary information

Supplement Material
