# Supplementary material for: Threat reversal learning and avoidance habits in generalised anxiety disorder
Source: Transl Psychiatry. 2022 May 31;12:216. doi: 10.1038/s41398-022-01981-3 (PMC9156703; doi:10.1038/s41398-022-01981-3)
Supplement: Supplementary file 1 — Supplement Material [file 41398_2022_1981_MOESM1_ESM.docx]

**Supplement**

**Additional trial by trial analyses of difference (CS+ minus CS-) scores (CS+ minus CS-) during Acquisition and Reversal**

We performed an additional trial-by-trial analysis of differential learning (CS+ minus CS-) SCR scores during acquisition and reversal to reveal time linked differences between the groups that could have been masked by averaging over trials. For this analysis we used each individual’s difference scores per trial (first CS+ minus first CS-; 2^nd^ CS+ minus 2^nd^ CS- etc.) for the 16 trials of each in in acquisition and reversal. This way we were able to show how differential learning per trial developed in each group (See Figure S1).

For this trial-by-trial analysis we used a repeated measures Anova with 2 stages (Acquisition and Reversal) and 16 difference scores for each stage analysed between groups. As Mauchly's test for sphericity was significant (p = 0.003) a Huyn-Feldt correction was applied (p = 0.935). There was a highly significant overall effect of group, indicating stronger differentiation over all trials in controls (F(52,1) = 24.5, p < 0.0001) and a significant 3-way interaction (stage by trials by group; F(14.02,729.2) = 1.764, p = 0.04).

**Trial by trial learning during Acquisition and Reversal using SCR difference scores**


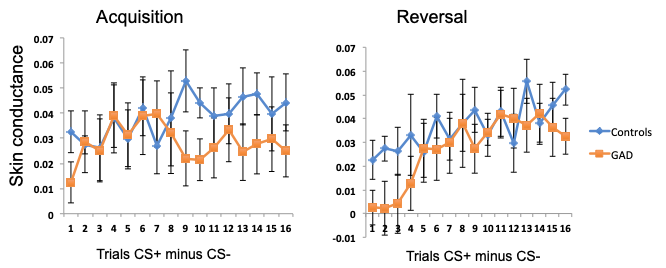


[**Figure S1** Depicts individual averaged trial by trial difference SCRs per group during acquisition and reversal. Error bars denote SEM.]

This additional analysis confirms that although early differential learning was not impaired overall in GAD patients, there were, in fact, changes in the strength of differential learning in GAD patients revealed by a trial by trial analysis. This lower strength in differential conditioning for GAD patients can be clearly observed in Figure S1 as reduced maintenance of differential SCRs during acquisition and slower adjustment of SCRs during reversal.

**Full ANOVA table for the Three-Way ANOVA (Learning stage, phase, skin conductance)**


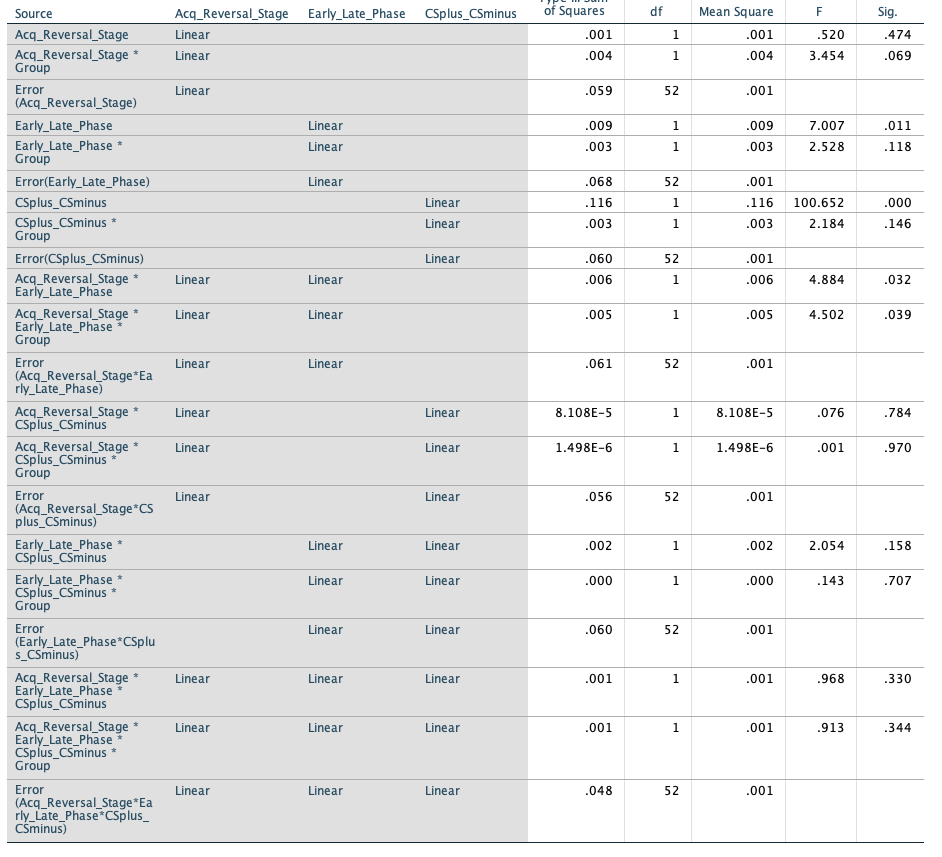


[**Table S1** Depicts the full Three-Way Anova from SPSS (Learning stage (Acquisition/Reversal, Phase (Early/Late), SCRs (CS+/CS-)).]
